# *In vitro* effect of fresh and frozen seminal plasma on the proliferation and viability of periodontal ligament fibroblasts

**DOI:** 10.5935/1518-0557.20260048

**Published:** 2026

**Authors:** Sara Valentina Oquendo Cano, Manuela González Muñoz, Mónica Alejandra Mahecha Peña, Jenniffer Puerta Suárez

**Affiliations:** 1 Pharmaceutical chemistry student, Reproduction Group, Department of Obstetrics and Gynecology, Faculty of Medicine, University of Antioquia, Medellín, Colombia; 2 Microbiologist and bioanalyst, university professor, Reproduction Group, Department of Obstetrics and Gynecology, Faculty of Medicine, University of Antioquia, Medellín, Colombia

**Keywords:** seminal plasma, cell migration, cell proliferation, polyamines

## Abstract

**Objective:**

To evaluate the effects of fresh and frozen seminal plasma, at three different concentrations, on the migration and viability of periodontal ligament fibroblasts.

**Methods:**

Semen samples from three healthy volunteers were processed to obtain seminal plasma, filtered (0.22 µm), and diluted to 2.5% and 5% (v/v) in DMEM. Aliquots were tested either fresh or after storage at -20°C for 8 days. Cell migration was evaluated by measuring wound closure in a wound-healing assay using photographs analyzed with ImageJ. Cell proliferation was assessed in triplicate at 6, 12, and 24 hours using the commercial CellTiter 96® AQueous Non-Radioactive Cell Proliferation Assay kit.

**Results:**

Across donors, frozen seminal plasma showed a higher mean percentage of wound closure at 48 hours than fresh seminal plasma; however, differences versus controls were not consistently significant, and viability tended to decrease after exposure to seminal plasma, particularly after freezing.

**Conclusion:**

Fresh and frozen seminal plasma did not promote cell migration or proliferation at the concentrations tested. The apparent trend toward greater migration with frozen samples requires confirmation in adequately powered studies and should be interpreted cautiously.

## INTRODUCTION

If not properly treated, oral tissue injuries may result in wounds or trauma that cause pain and discomfort. They may be caused by accidents, surgical procedures, physical trauma, exposure to extreme temperatures, or chemical trauma (Thone *et al*., 2023). These injuries are common in the general population, with a prevalence ranging from 5% to 65%, and the most common type is traumatic (Toro-Alzate *et al*., 2024). They may have diverse causes, including sexual practices such as fellatio. In this context, oral sex has become one of the most common sexual practices in recent years, with reported frequencies of 85% among heterosexual individuals (Copen *et al*., 2016) and 72% among homosexual individuals (Bjekic *et al*., 2018; Rosenberger *et al*., 2011). Oral sex is also associated with lower condom use (Ballester-Arnal *et al*., 2022; Gómez-Melasio *et al*., 2024) and a higher risk of infection transmission, since semen is recognized as a vehicle for the transmission of infections, as widely documented in the literature (Guerrero-Hurtado *et al*., 2018; Puerta Suárez & Cardona Maya, 2016; 2024; Puerta Suarez *et al*., 2017; Restrepo Arenas *et al*., 2021; Saldarriaga López *et al*., 2024; Velásquez Rivera *et al*., 2022; Velásquez Rivera *et al*., 2018; Zuleta-González *et al*., 2019). Aside from its role in the transmission of infections, semen contains substances such as polyamines, which are involved in wound-healing processes (Ito *et al*., 2021).

Given this context, we sought to evaluate the in vitro effects of fresh and frozen seminal plasma at three concentrations on PLF cells (periodontal ligament fibroblasts) as a model for understanding other potential effects of oral sex on the physiology of cells in the oral cavity.

## MATERIALS AND METHODS

Ethics and donors: Semen samples were obtained from three healthy volunteers aged 18-30 years who were asymptomatic for urogenital infections and who had avoided the use of lubricating ointments or creams on their genitals for 24 hours before sample collection (World Health Organization, 2021). For this study, participants had no history of sexually transmitted infections or related symptoms and agreed to participate by signing an informed consent form. No tests were performed to detect microorganisms in the samples; however, they were handled in accordance with standard biosafety precautions for biological samples. Although screening study participants for sexually transmitted infections is important, the absence of these microorganisms in semen samples would not rule out the presence of other microorganisms that may be part of the genitourinary tract microbiota and therefore may be transferred to semen. Thus, it is difficult to completely eliminate the impact of microorganisms in this type of study. Consequently, both symptomatic and asymptomatic infectious processes, as well as the presence of microorganisms in the male urogenital tract, may affect the proper interpretation of these results. Sample collection was carried out within the framework of an ongoing project for the detection of sexually transmitted infections in university students, approved by the Ethics Committee of the Institute of Medical Research of the Faculty of Medicine of the University of Antioquia (Act 048, May 11, 2023). Participants were asked whether they were willing to donate the remainder of the sample obtained for the present study, and they provided written approval. Sample collection: Samples were collected by masturbation after 2 to 5 days of sexual abstinence and placed in sterile containers. Semen quality was assessed according to the World Health Organization (WHO) guidelines in the 2021 manual for the examination and processing of human semen (World Health Organization, 2021). No screening for sexually transmitted infections was performed as an inclusion criterion for semen donors in this study. The samples were then centrifuged at 1500 rpm (750 × g) for 5 minutes at 25°C, diluted in DMEM (Gibco, NY, USA), and filtered through a 0.22-µm filter (Biologix, Jinan, China). Final seminal plasma concentrations of 2.5% and 5% were prepared for each well. Because semen samples exhibit substantial biological variation, the same sample from each volunteer was used for assays with both fresh and frozen seminal plasma. Therefore, after centrifugation, dilution, and filtration, each sample was divided into aliquots. The samples were aliquoted to avoid repeated freeze-thaw cycles; thus, all samples were thawed only once. This freezing process was not intended as cryopreservation; rather, the samples were stored at -20°C to evaluate whether freezing-related temperature changes could improve wound healing, based on the theory of polyamine precipitation. However, polyamines were not quantified in the samples. The assay was performed in triplicate with each sample using both fresh and frozen seminal plasma. Frozen samples were stored at -20°C for 8 days until the assay was repeated; before use, they were completely thawed at room temperature for 10 minutes before being placed in contact with the cells seeded in the culture dishes.

Cell migration assessment: PLF cells were cultured in DMEM supplemented with 10% (v/v) fetal bovine serum (FBS; Gibco, NY, USA) and 1% antibiotics (100 U/mL penicillin, 100 µg/mL streptomycin [Gibco, NY, USA], and 100 U/mL gentamicin [Genfar, Bogotá, Colombia]) at 37°C and 5% CO_2_ until they reached 90% confluence. The cells were then transferred to 24-well plates at a concentration of 160,000 cells/mL. The wound assay was performed according to modifications of a previously proposed protocol (Cappiello *et al*., 2018; Ito *et al*., 2021). Unidirectional wounds were created with a sterile micropipette tip, after which the corresponding experimental samples were added at concentrations of 2.5% and 5% seminal plasma. As a growth control, cells were incubated in medium supplemented with 10% FBS, and the effect of seminal plasma on the cell line was also compared with that of a 5% Fitostimoline Gel (Euroetika, Medellín, Colombia), a wound-healing gel for mucosa and skin. Each well was monitored and photographed, and cell migration was measured at 0, 6, 24, and 48 hours. One photograph was obtained from each well at each incubation time in a random field, without standardization, and the test was performed in triplicate for samples from each of the three participants. The photographic records used to assess cell migration were analyzed with ImageJ software (version 1.54; NIH, MD, USA). Image analysis included measuring wound width in micrometers; however, to facilitate interpretation, these data were normalized. To express the percentage of wound closure, the wound width in each well at time zero was measured in micrometers and defined as 100% of the wound. The following formula was then applied at the different time points: percentage of wound closure = (wound size in micrometers at the evaluated time point × 100) / wound size at time 0.

Evaluation of cell proliferation: To evaluate the effects of fresh and frozen seminal plasma on cell proliferation, as well as the synthesis and accumulation of polyamines at low temperatures, PLF cells were transferred to 96-well plates at 100 µL/well, and dilutions of the three samples were added. The growth control and the commercial cream were incubated at 37°C and 5% CO_2_, and cell viability was then assessed using the commercial CellTiter 96® AQueous Non-Radioactive Cell Proliferation Assay kit (Promega Corporation, Madison, WI, USA) according to previously described recommendations (Kamiloglu *et al*., 2020).

Statistical analysis: Semen samples from three volunteers were included and divided for assays using fresh and frozen plasma because of the high biological variability of semen samples. Three independent assays were performed to evaluate cell migration and proliferation with fresh plasma, and three independent assays were performed for both outcomes with frozen plasma, since the sample from each volunteer used for the fresh and frozen evaluations was tested on different days but under the same conditions described in the Methods. In both experiments, the procedure was performed in triplicate (three wells per sample). The assays for fresh and frozen samples were conducted one week apart. Results were expressed as percentages of wound closure and cell viability, respectively. Descriptive statistics and ANOVA were used to evaluate both cell migration and proliferation, and the Kruskal-Wallis test was used for cell viability, using Prism 9.0 (GraphPad Software, San Diego, CA).

## RESULTS

**Figure 1:**
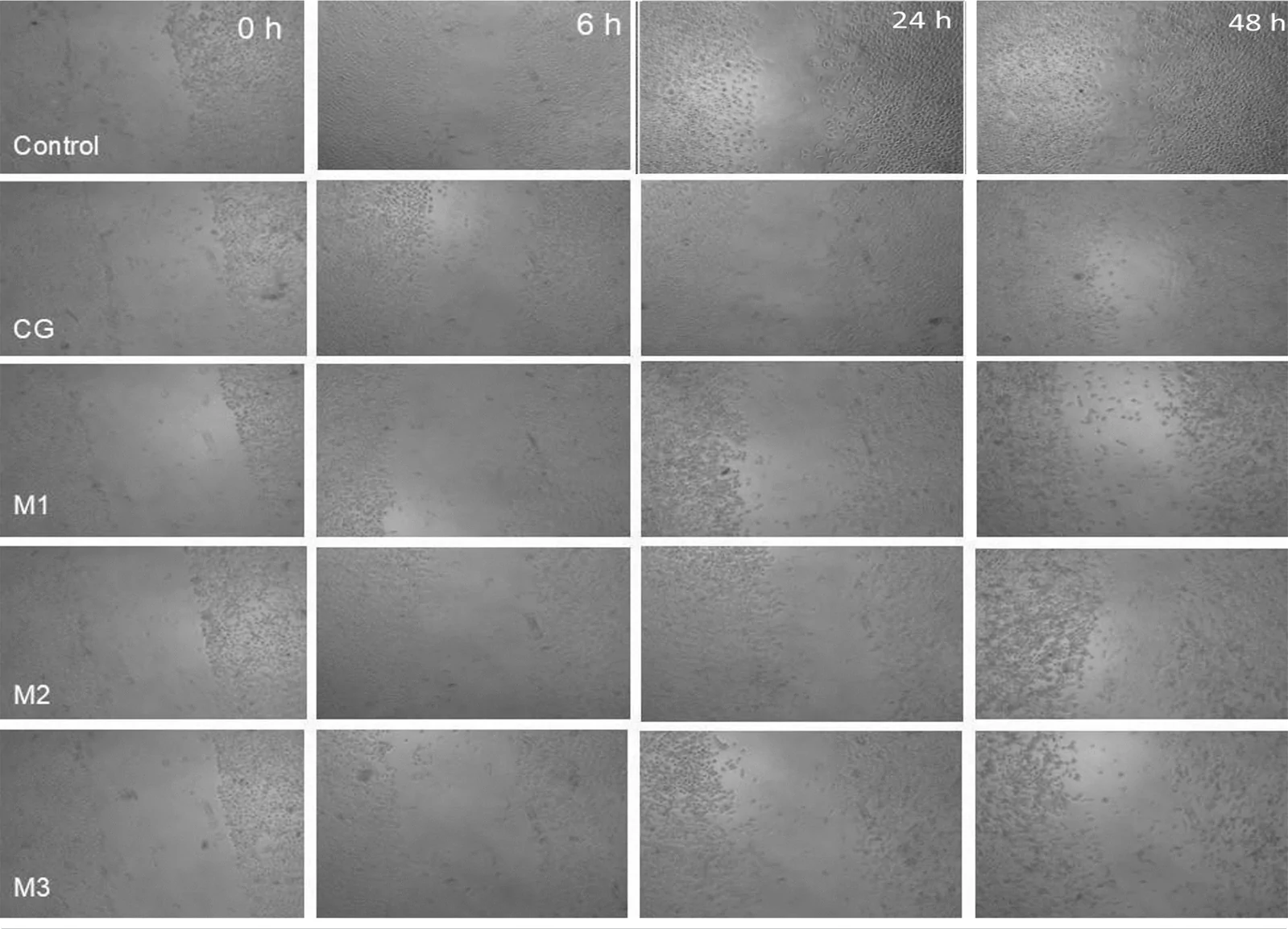
Representative images of the wound assay for the growth control (Control), commercial gel (CG), seminal plasma sample 1 (M1), seminal plasma sample 2 (M2), and seminal plasma sample 3 (M3) at 0, 6, 24, and 48 hours.

**Figure 2:**
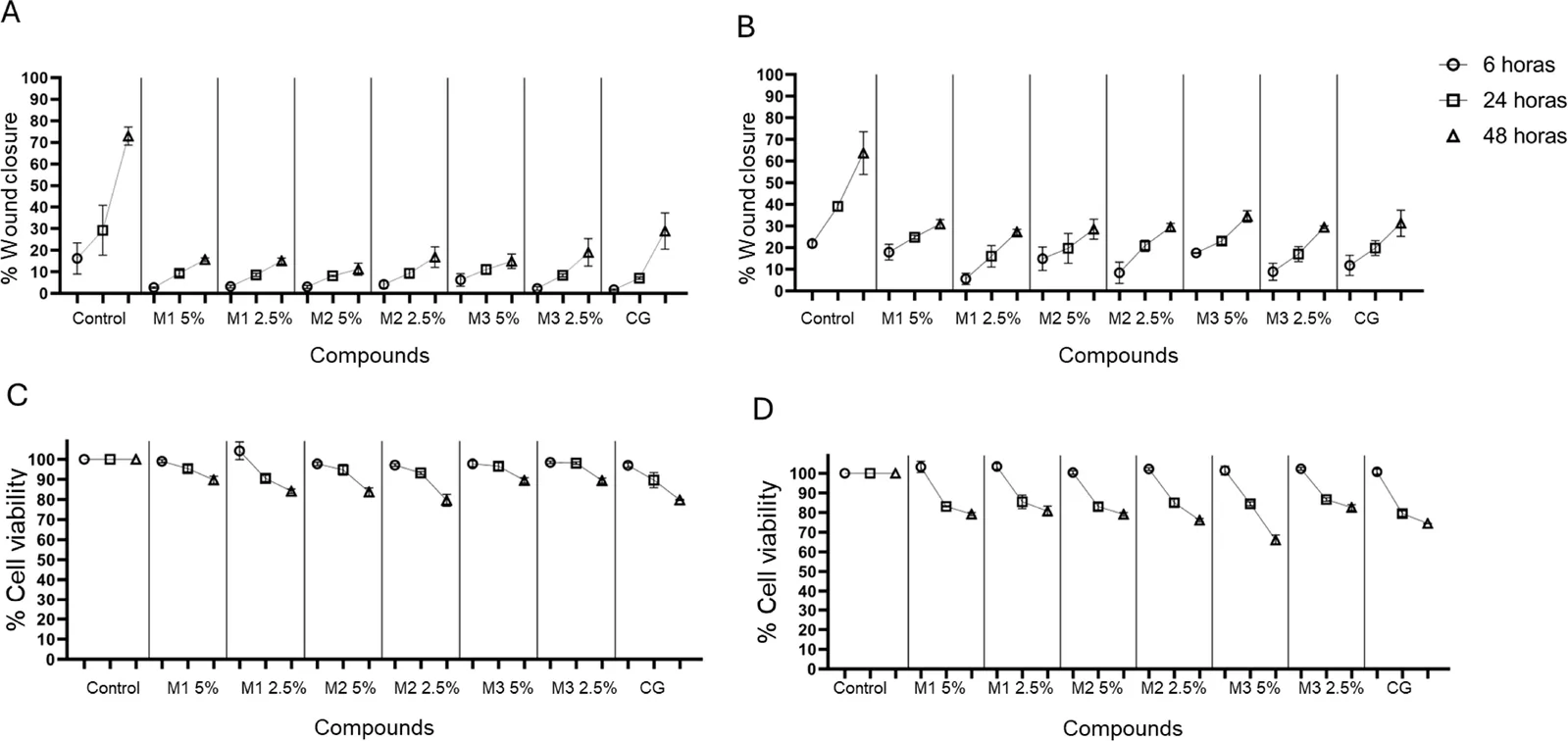
Evaluation of cell migration (wound assay) and cell viability in the PLF cell line for the growth control (Control), commercial gel (CG), seminal plasma sample 1 (M1), seminal plasma sample 2 (M2), and seminal plasma sample 3 (M3) at concentrations of 2.5% and 5% at 0, 6, 24, and 48 hours. A, fresh seminal plasma; B, frozen seminal plasma. C, fresh seminal plasma cell viability; D, frozen seminal plasma cell viability.

The volunteers’ semen samples were evaluated to verify that they met the lower reference limits for seminal parameters established in the WHO manual for the examination and processing of human semen (Table 1). A wound assay was performed using the PLF cell line, comparing wound closure in the presence of three seminal plasma samples, both fresh and frozen, at two concentrations (2.5% and 5%); a growth control (Control; medium with 10% FBS); and a positive control consisting of a commercially available gel (CG; Fitostimoline Gel 15 g). Photographic monitoring was performed at 0, 6, 24, and 48 hours, showing that fresh seminal plasma promoted wound closure of at least 2.4% in all cases, whereas frozen seminal plasma promoted closure of at least 29.1% in all cases. At 48 hours, frozen seminal plasma showed a higher average closure (30%) than fresh seminal plasma (15%), demonstrating behavior similar to that of the Fitostimoline healing gel (Figure 1). The wound-closure data for both fresh and frozen samples are presented in Table 2. In both cases, the average closure percentages were lower than those of the control. Cell viability decreased under all conditions compared with the control, with frozen seminal plasma samples showing the lowest viability, similar to the findings for the Fitostimoline healing gel (Figure 2).

**Table 1:** Sperm parameters

Seminal parameter (unit)	Sample 1	Sample 2	Sample 3
Sperm Mobility (%) a: rapid progressive mobiles b: slow progressive mobiles c: non-progressive mobiles d: immobile	38 28 8 24	12 47 3 36	36 28 9 24
Viability (%)	85	86	80
Concentration (millions of sperm/mL)	376	51	165

**Table 2:** Wound closure percentage and standard deviation with fresh and frozen samples. Growth Control (Control), Commercial Gel (CG), seminal plasma sample 1 (M1), seminal plasma sample 2 (M2), and seminal plasma sample 3 (M3) at 0, 6, 24, and 48 hours

Condition	Sample	Time	Percentage of wound closure			Average	Standard deviation
Fresh samples	Control	6 h	19.4	2.5	26.9	16,3	12,5
		24 h	33.3	7.6	47	29,3	20,0
		48 h	78.1	64.7	76	72,9	7,2
	M1 2.5%	6 h	3.3	3	2.1	2,8	0,6
		24 h	9.6	11.5	7	9,4	2,3
		48 h	15.6	17.2	14.3	15,7	1,5
	M1 5%	6 h	2.4	4.5	2.9	3,3	1,1
		24 h	7.2	9.6	9.1	8,6	1,3
		48 h	17.8	13.6	13.9	15,1	2,3
	M2 2.5%	6 h	4.5	2.3	2.5	3,1	1,2
		24 h	8.7	7.5	8.4	8,2	0,6
		48 h	14.6	5.6	13.7	11,3	5,0
	M2 5%	6 h	7.3	2.2	3	4,2	2,7
		24 h	13.9	6.5	7.6	9,3	4,0
		48 h	26.3	11.4	12.9	16,9	8,2
	M3 2.5%	6 h	5.1	2	11.8	6,3	5,0
		24 h	11.9	6.9	14.6	11,1	3,9
		48 h	16.3	8.6	19.9	14,9	5,8
	M3 5%	6 h	4	1.3	1.9	2,4	1,4
		24 h	7.1	9.6	8.6	8,4	1,3
		48 h	12.7	31.8	12.7	19,1	11,0
	CG	6 h	2	2.3	1.1	1,8	0,6
		24 h	7.2	8	6.2	7,1	0,9
		48 h	42.3	31	13.5	28,9	14,5
Frozen samples	Control	6 h	18.7	23.8	23	21,8	2,7
		24 h	39.4	42.5	35.1	39,0	3,7
		48 h	44.9	67.5	78.6	63,7	17,2
	M1 2.5%	6 h	22.9	10.8	20	17,9	6,3
		24 h	26.4	24.7	23.2	24,8	1,6
		48 h	31.9	26.8	33.8	30,8	3,6
	M1 5%	6 h	0.8	9.6	6.2	5,5	4,4
		24 h	6.7	23.6	17.8	16,0	8,6
		48 h	25	29.4	27.1	27,2	2,2
	M2 2.5%	6 h	24.7	6	14	14,9	9,4
		24 h	28.6	6.2	24.2	19,7	11,9
		48 h	31.7	19.4	34.4	28,5	8,0
	M2 5%	6 h	3.8	18.2	3.1	8,4	8,5
		24 h	16.4	25.5	20.6	20,8	4,6
		48 h	28.6	32.8	27.3	29,6	2,9
	M3 2.5%	6 h	17.2	17.9	17.4	17,5	0,4
		24 h	20.1	24.4	24.7	23,1	2,6
		48 h	29.2	37.5	36.6	34,4	4,6
	M1 5%	6 h	3.6	16.6	6.3	8,8	6,9
		24 h	15.6	23.5	11.9	17,0	5,9
		48 h	30.7	28.6	28.9	29,4	1,1
	CG	6 h	8.1	6.2	21.1	11,8	8,1
		24 h	19.5	14	25.8	19,8	5,9
		48 h	33.9	19.7	40.2	31,3	10,5

## DISCUSSION

The oral environment is currently susceptible to frequent exposure to semen because of the increase in sexual practices such as oral sex or “snowballing,” in which seminal plasma may be deposited in the oral cavity. This leads to direct contact of this biological fluid with oral tissues and therefore creates a risk associated with sexually transmitted infections (STIs), considering that receptive oral sex increases exposure of the oropharyngeal mucosa to the most common type of infection, human papillomavirus, and therefore to a higher risk of oropharyngeal cancer (Wierzbicka *et al*., 2021). At the same time, oral sex may also be associated with oral or pharyngeal syphilis, pharyngitis due to Chlamydia trachomatis, and herpes simplex virus infection, all of which are widely reported in the literature (Fernández-López & Morales-Angulo, 2017). Within the oral cavity, the periodontal ligament fibroblast (PLF) is the primary ligament cell surrounding the tooth root and contributes to the maintenance of soft and hard tissues as well as periodontal immune balance (Huang *et al*., 2024). One of its main functions is the formation of connective tissue fibers, specifically collagen and elastin. However, it can also synthesize and phagocytose collagen and extracellular matrix components during connective tissue remodeling, and periodontal ligament fibroblasts play a decisive role in the healing of oral wounds, especially in the context of traumatic periodontal ligament injuries. This specialized connective tissue is essential for the integration and support of teeth in the alveolar bone, and its response to injury is vital for tissue recovery (Gómez, 2006).

Semen has been widely studied as a biological fluid in scientific research. This has led to the identification of compounds known as polyamines, which have been correlated with cell migration processes, with higher levels observed in rapidly growing tissues and under hormonal stimulation (Pegg, 2016). Polyamines present in semen, such as spermidine, spermine, and putrescine, are molecules synthesized by mammals that function in cell growth and differentiation, cell cycle regulation, gene expression, and signal transduction in various organisms. In addition, they are involved in pathophysiological processes such as carcinogenesis (Agostinelli, 2020). At the same time, their metabolism is associated with biological functions such as genetic regulation, cell-cell interactions, cell proliferation and differentiation, immune response, and apoptosis (Sagar *et al*., 2021). Based on the above, in this study we evaluated the effect of seminal plasma on the migration and proliferation of PLF cells using fresh semen samples, which were tested within no more than 2 hours of collection, and frozen semen samples, which were kept at -20°C for 8 days until use and subsequently brought to room temperature before being added to the cells. The potential of semen as an agent associated with cell migration during wound healing has been suggested in relation to the presence of polyamines (Pegg, 2016). It has also been reported that, under freezing conditions, polyamines may be actively synthesized because low-temperature stress promotes the expression of genes associated with polyamine oxidase, thereby maintaining polyamine levels through synthesis and accumulation induced by freezing stress (Li *et al*., 2024; Yang *et al*., 2023).

The literature has shown that the main components of the periodontal ligament fibroblast extracellular matrix include fibrillar collagens, whereas nonfibrillar collagens are present in smaller proportions. These high collagen turnover rates provide tissue shape, connectivity, and strength (Kaku & Yamauchi, 2014). This compound could also be involved in the biological processes of spermidine, as reported by Kim *et al*. (2021), who found that this polyamine is associated not only with autophagy and cell viability but also with increased collagen and elastin expression in the skin. However, additional research is needed to determine whether this behavior can be extrapolated to the oral cavity.

Along these lines, studies such as that of Wang *et al*. (2023) have shown that the in vitro use of spermidine in biomaterials, such as medical implants, reduces inflammation and foreign-body reactions. In addition, Ito *et al*. (2021) reported that systemic and topical administration of this polyamine accelerates cutaneous wound healing by inducing the production of proinflammatory cytokines in the wound area, possibly accelerating wound closure. However, our study differs from previous reports by using PLF cells, which allow evaluation of this apparent phenomenon specifically in the context of oral wounds resulting from oral sexual practices or prior to them, an area that remains insufficiently explored in scientific research.

In general, our results showed approximately 30% wound closure at 48 hours with frozen seminal plasma, compared with approximately 15% with fresh seminal plasma, although there were no statistically significant differences (data not shown). Although we cannot demonstrate that polyamines are responsible for the effects observed in the present study, freezing conditions may have induced changes in the effect of seminal plasma (32, 33), thereby affecting PLF cell migration compared with fresh samples.

When considering the findings of Lim *et al*. (2018), one promising mechanism to investigate is the role of AMD1 in the metabolism of polyamines present in semen, because it regulates their proportions. In its absence, spermine levels and the speed of the cell migration process decrease, but this effect can be reversed by adding more spermine. This observation suggests that, in future studies, higher concentrations of seminal plasma might increase the percentages observed for cell migration and proliferation. According to the results obtained, other factors not directly evaluated in the present study may also have influenced the findings, such as the biological variability of each semen sample, which could significantly affect its ability to promote healing. Additional factors, including sample quality, freezing technique, donor diet and habits, and the exact cell culture conditions, may also modify the results. Therefore, the high variability in the fresh-sample data, as reflected by the standard deviations, constitutes a limitation of the present study and restricts our ability to draw definitive conclusions regarding the effect of semen samples on the migration of PLF cells. Nevertheless, additional studies are required to define more precisely the roles of semen and the polyamines it contains, as well as the molecular mechanisms underlying the effects of freezing on cell migration and wound healing. It should be noted that, although the data suggest an overall trend toward increased wound closure over time and variability in both fresh and frozen semen samples, no statistically significant differences were observed between the semen-treated and control groups, except between the control group at 48 hours and sample 1 at 2.5% at 6 hours (*p*=0.0459) in the frozen semen samples. Finally, it is important to clarify that this study arose from scientific curiosity, and the results must be interpreted with caution. The main reasons for this cautious interpretation are the biological variability of semen samples and their composition, especially the concentration of polyamines, which was not quantified. The use of fresh seminal plasma and its comparison with the same sample after freezing at -20°C for several days was based on the assumption that polyamine crystals precipitate, a claim we were unable to confirm in this study. Although this is a novel investigation, it has multiple limitations, particularly with respect to the biochemical measurements needed to quantify polyamine crystals. In future studies, similar tests should be performed using commercial polyamines (spermine and/or spermidine), which can be administered at specific concentrations or at least quantified when obtained from seminal samples.

## CONCLUSIONS

Fresh and frozen seminal plasma did not promote cell migration or proliferation at the concentrations used in PLF cells. The apparent trend toward greater migration with frozen samples requires confirmation in adequately powered studies and should be interpreted cautiously.
